# re-TAMD: exploring interactions between H3 peptide and YEATS domain using enhanced sampling

**DOI:** 10.1186/s12900-018-0083-6

**Published:** 2018-04-03

**Authors:** Gilles Lamothe, Thérèse E. Malliavin

**Affiliations:** 1 Unité de Bioinformatique Structurale, UMR CNRS 3528 and Institut Pasteur, Paris, France; 2 Université Denis Diderot Paris 7, Paris, France

**Keywords:** Protein/peptide interaction, Enhanced sampling, TAMD

## Abstract

**Background:**

Analysis of preferred binding regions of a ligand on a protein is important for detecting cryptic binding pockets and improving the ligand selectivity.

**Result:**

The enhanced sampling approach TAMD has been adapted to allow a ligand to unbind from its native binding site and explore the protein surface. This so-called re-TAMD procedure was then used to explore the interaction between the N terminal peptide of histone H3 and the YEATS domain. Depending on the length of the peptide, several regions of the protein surface were explored. The peptide conformations sampled during the re-TAMD correspond to peptide free diffusion around the protein surface.

**Conclusions:**

The re-TAMD approach permitted to get information on the relative influence of different regions of the N terminal peptide of H3 on the interaction between H3 and YEATS.

## Background

Docking of small ligands, chemical compounds or peptides, on proteins is quite an important problem encountered in various fields of structural bioinformatics, from drug design studies [1] to analysis of functional networks within the cell [2].

The efficiency of the docking depends on two ingredients: (i) the availability of a reliable score to select the ligand poses corresponding to the largest experimental affinity, (ii) the ability to efficiently sample the relative positions of the ligand within a given pocket or on the protein surface. Several possibilities exist for calculating scores: absolute free energy of interaction [3], QM/MM (quantum mechanics/molecular dynamics) based approaches [4] or rescoring of obtained poses [5].

Concerning the point (ii), one should notice that most of the past virtual screening approaches have focused on the docking of the ligand on a pre-defined pocket [6–8]. Nevertheless, several methods [9–16] were then developed to use molecular dynamics simulations to explore the protein surface without being limited to a given spot. The development of such approaches is justified by the importance of detecting new pockets on protein surfaces: these pockets have been used for lead optimization [17, 18] or, to overcome resistance problems [19, 20].

In the context of molecular dynamics simulations, two main types of exploration approaches have been proposed. Firstly, molecular dynamics trajectories are recorded [9–12] on the studied protein solvated with a mixture of water and various polar and apolar small compounds representing different types of interactions. These trajectories are then analyzed to determine the most populated positions of the compounds on the protein surface, allowing to predict surface hot-spots [11, 21] that should then be targeted by virtual screening studies.

Secondly, other methods have taken advantage of the growing efficiency of enhanced sampling approaches, such as metadynamics [22]. Two types of methods have been proposed: the funnel metadynamics for exploring the conformations of a ligand on a pocket, loosely-defined by a funnel [13, 14], and metadynamics approaches that allow the exploration of the receptor surface by the ligand [15, 16]. Both methods are effective, and permit converged estimation of the interaction free energy, but with a large computational cost.

We propose here an approach, re-TAMD (reconnaissance-TAMD) for exploring the protein surface based on the temperature-accelerated molecular dynamics (TAMD) [23, 24], an enhanced sampling approach which proved its efficiency on various biological systems [25–30]. Similarly to TAMD, re-TAMD requires less computational power than metadynamics-derived approaches. Although the re-TAMD approach proposed here does not provide a formal picture of free-energy surface, it has the advantage of being specific to the studied system, unlike the methods based on fragment probes [9–12, 16].

We applied this approach to the study of interactions involving post translational modifications (PTMs), which frequently occur in proteins for regulatory purposes. PTMs play an important role in histones [31], proteins which are wrapped by base pairs of DNA forming the nucleosome complex [32] and are involved in gene expression [33–35] and chromatin dynamics [36, 37].

Recent studies have shown that lysines modified by acylations - a class of PTMs - interact with the YEATS domain (named after the Yaf9, ENL, AF9, and Sas5 family), a strongly conserved domain found in several epigenetics reader proteins across many species [38–42]. The study of interactions between PTMs and epigenetic readers is largely motivated by findings that show links between readers and cancer cell proliferation [43–45]. In the present work, we applied the re-TAMD approach to the study of the interaction between AF9’s YEATS domain and the H3 histone N-t tail’s acetylated lysine 18 (acK18). Using enhanced sampling, we looked at the influence of the peptide length on the interaction with the protein.

## Methods

### Studied systems, collective variables and trajectories

**Fig. 1:**
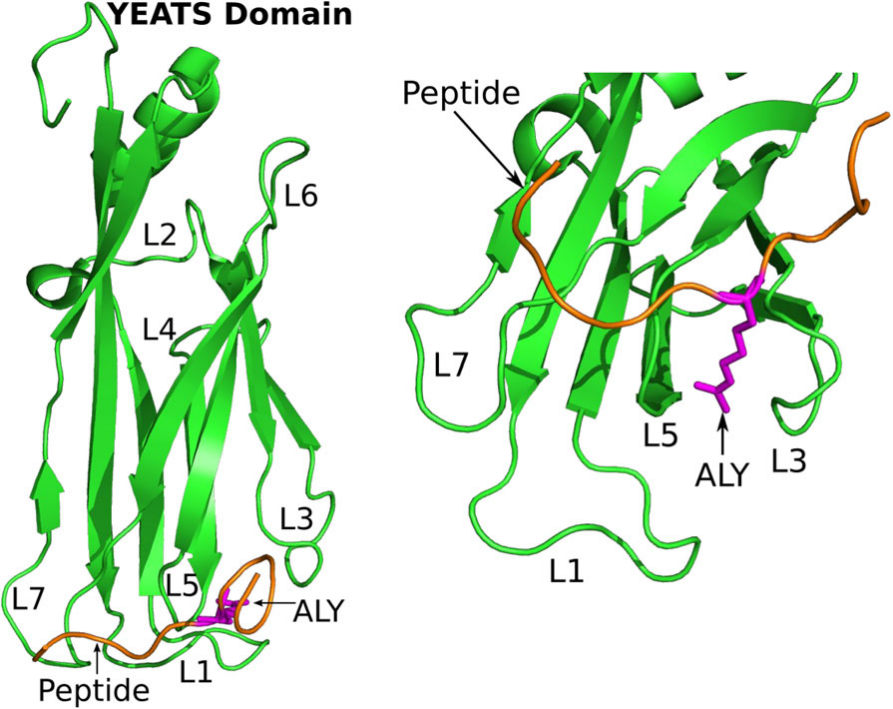
YEATS–Peptide complex. PDB 2NDF. Green: human AF9 YEATS. Orange: 13-residue fragment of H3 histone’s N terminal tail. Magenta: acK18

Several systems were prepared using selected atoms from the first model of the NMR (Nuclear Magnetic Resonance) structure (PDB entry: 2NDF) [38] (Fig. 1). Each molecule or complex was solvated in a water box using the Amber 14’s LEaP program [46] and the Amber ff03 force field [47] along with a specific parameter file to account for residue acK18 (called ALY) [48]. The systems were then minimized, thermalized, and equilibrated using NAMD 2.9b2 [49].

The following systems were studied: (i) the complexes between the protein YEATS and the peptides 12-24 (pt13), 15-21 (pt7) and 17-19 (pt3) from the N terminal tail in histone H3, (ii) the YEATS domain in the absence of the peptides and (iii) the isolated peptides pt13, pt7 and pt3. The studied systems along with the launched simulations are given in Table 1.

**Table 1:** Conditions used for the MD and re-TAMD simulations

Name	Duration	Type/number	Number of waters	Number of atoms	Solute
	(ns)	of counterions			
MDYpt13	100	Cl-/8	10261	33331	YEATS/pt13
MDYpt7	100	Cl-/6	11615	37309	YEATS/pt7
MDYpt3	100	Cl-/6	11636	37319	YEATS/pt3
MDYapo	100	Cl-/5	10166	32838	YEATS
MDpt13	100	CL-/3	2949	9055	pt13
MDpt7	100	Cl-/1	2208	6748	pt7
MDpt3	100	Cl-/1	1751	5324	pt3
TAMDYpt13	100	Cl-/8	10261	33331	YEATS/pt13
TAMDYpt7	100	Cl-/6	11615	37309	YEATS/pt7
TAMDYpt3	100	Cl-/6	11636	37319	YEATS/pt3

pt13: peptide residues 12-24. pt7: peptide residues 15-21. pt3: peptide residues 17-19

### Description of re-TAMD

**Fig. 2:**
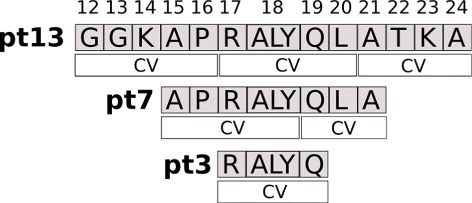
Peptides used in the simulations. The collective variables (CVs) are geometrical centers of the selected residues. They were used for the re-TAMD simulations

The temperature-accelerated molecular dynamics (TAMD) approach is an enhanced sampling approach, based on the parallel evolution of the protein coordinates ***x*** in a classical MD simulation (Eq. 1) and of the target values ***z*** for the collective variables (CV) *θ*_*α*_(***x***) (Eq. 2):

1 $$ {} \begin{aligned} M\ddot{\boldsymbol{x}} &=-\gamma \dot{\boldsymbol{x}}-\nabla_{x} V(\boldsymbol{x}) -\kappa \sum_{\alpha=1}^{N} \left(\theta_{\alpha}(\boldsymbol{x}\right) &- z_{\alpha})\nabla_{x} \theta_{\alpha}(\boldsymbol{x})\\[-4pt] \quad &\quad~+ \sqrt{2 M\gamma \beta^{-1}}\, \boldsymbol{\eta}^{x}(t) \end{aligned}  $$

2 $$ \bar \gamma \dot{\boldsymbol{z}} = \kappa \left(\boldsymbol{\theta}(\boldsymbol{x}) - \boldsymbol{z}\right) + \sqrt{2 \bar\gamma \bar \beta^{-1}}\, \boldsymbol{\eta}^{z}(t)  $$

where ***x*** are the physical variables (atomic coordinates) of the system, *θ*(***x***) are the current values of the collective variables and ***z*** the ever evolving target values of the collective variables. Several sets of collective variables were used on the peptides only (Fig. 2). *M* is the mass matrix, *V*(***x***) is the empirical classical potential of the system, ***η***^*x*,*z*^(*t*) are white noises (i.e. Gaussian processes with mean 0 and covariance $<\eta ^{p}_{\alpha }(t)\eta ^{p}_{\alpha '} (t')> = \delta _{\alpha \alpha '} \delta (t-t')$, with *p*=***x***,***z***), *κ*>0 is the so-called spring force constant, $\gamma, \bar \gamma >0$ are friction coefficients of the Langevin thermostats, *β*^−1^=*k*_*B*_*T*, $\bar \beta ^{-1}=k_{B}\bar T$ with *k*_*B*_ the Boltzmann constant and $T, \bar T$ the temperatures.

Equations 1 and 2 describe the motion of ***x*** and ***z*** under the extended potential

3 $$ U_{\kappa}(\boldsymbol{x},\boldsymbol{z}) = V(\boldsymbol{x}) + \tfrac12 \kappa \left\| \boldsymbol{\theta}(\boldsymbol{x}) - \boldsymbol{z} \right\|^{2}.  $$

It was shown in [23] that by adjusting the parameter *κ* so that ***z***(*t*)≈***θ***(***x***(*t*)) and the friction coefficient $\bar \gamma $ so that the ***z*** move slower than ***x***, one can generate a trajectory ***z***(*t*) in *z*-space which effectively moves at the artificial temperature $\bar T$ on the free energy hyper-surface *F*(***z***) defined at the physical temperature *T*. Then, using $\bar T > T$ in Eq. 2 accelerates the exploration of the free energy landscape by the ***z***(*t*) trajectory, as energy barriers can be crossed more easily.

The TAMD approach was implemented in NAMD using a tcl script [25–27]. The friction coefficient, *γ*=2 ps^−1^, and the physical thermal energy, *β*^−1^=0.6 kcal/mol, are the parameters of the conventional Langevin thermostat, allowing to obtain a simulation temperature of 300 K. The restraint force constant is set to *κ*=100 kcal/(mol.Å^2^).

Along the re-TAMD trajectories, the artificial friction $\bar {\gamma }$ of the Langevin thermostat attached to the collective variables was set as a constant equal to 0.02 ps ^−1^, whereas the artificial thermal energy $\bar {\beta }^{-1}$ was varied continuously depending on the smallest distance *min*(*D*) between the H3 peptide and the YEATS domain.

4 $$ \bar{\beta}^{-1} = \frac{k}{min(D)} + h  $$

where *h* and *k* values are given in Table 2.

**Table 2:** Enhanced sampling parameters used for each TAMD simulation

System Name	Parameter c	Parameter k	Parameter h	Number of CVs	Numbers of atoms
		(Å.kcal/mol)	(kcal/mol)		defining each CV
TAMDYpt13	0.02	40	10	3	68
TAMDYpt7	0.02	30	10	2	60
TAMDYpt3	0.7	30	10	1	67

In order to keep the peptide close to the YEATS domain, at each simulation step, the distances $D_{i}^{new}$ measured between the new target values of the *i*-th CV and the YEATS domain were compared to the corresponding previous distances *D*_*i*_. The following soft-ratcheting criterion [29, 50, 51] was used for accepting or rejecting the new target values of the peptide’s collective variables. If at least one distance $D_{i}^{new}$ is smaller than the corresponding distance *D*_*i*_ all of the new target values are accepted as the current target values. Otherwise, the new values are accepted with a probability of *min*(1,[*f*_1_*f*_2_⋯*f*_*N*_]) where:

5 $$ f_{i} = exp\left[-\left(D_{i} - D_{i}^{new}\right)^{2}\right]\big/c D_{i}^{2}  $$

where *c* is the restraint coefficient that determines how strict is the distance restraint. The *c* values are given in Table 2.

For the native peptide, the values of *h*, *k* and *c* were chosen as values for which a complete exploration of the YEATS surface is performed by the peptide. As the peptides pt7 and pt3 have a smaller mass, they flow away from the protein surface, not allowing a satisfying exploration of the protein surface. So, the parameter *k* was decreased and the parameter *c* increased (Table 2) to prevent these peptides from separating from the protein.

The CPU time necessary to record one re-TAMD trajectory on the complex between the YEATS domain and the H3 peptide is between 10 and 17 days on computers with 16 cores, and GeForce GTX GPUs, using the CUDA version of NAMD 9.2b2.

### Analysis of trajectories

The atomic interactions, hydrophobic and polar, between the peptides and the protein, were analyzed by calculating the number of proximities (distance smaller than 4 Å) between polar/hydrophobic groups present in the peptide and in the protein throughout the trajectory. This analysis was performed using a python script based on the MDAnalysis module [52]. The number of inter-atomic contacts was rescaled between 0 and 1 for each trajectory. The number of contacts per residue was determined as the sum of atomic contacts involving each residue divided by the largest contact value. The partial charges used for this script were taken from the AMBER ff03 force field [47]; partial charges with absolute values smaller than 0.2 were considered to correspond to hydrophobic groups. The (*ϕ*,*ψ*) distributions were also calculated using MDAnalysis. The protein surfaces were calculated using PyMol [53].

## Results

An analysis of the acK18 position with respect to the residues of the native pocket (Table 3) reveals that, along the MD trajectories with the three different peptides, all acK18 display similar proximity with respect to most of the residues, which means that no peptide dissociation from the native site is observed. Nevertheless, pt7 and pt3 move apart from W35, I85 and L109, and get closer to H59 and S61, which is the unsurprising sign of a slight destabilization. On the other hand, during the re-TAMD trajectories, acK18 dissociates from its native binding site, which proves that the peptide moves away from this site.

**Table 3:** Percentage of frames during which a given residue of the acK18 binding site is in contact with acK18 (contact distance <4 Å) along the MD and TAMD trajectories

Pocket residue	MDYpt13	MDYpt7	MDYpt3	TAMDYpt13	TAMDYpt7	TAMDYpt3
	(%)	(%)	(%)	(%)	(%)	(%)
F31	89.2	98.4	88.1	1.0	0.5	5.6
W35	45.1	6.6	4.9	0.0	0.0	0.1
H59	55.7	99.9	99.9	5.0	1.3	9.7
S61	33.1	95.4	97.3	2.6	0.8	7.2
F62	98.2	100.0	99.9	0.8	0.3	5.5
S79	65.2	57.4	57.0	0.0	0.1	0.3
G80	99.6	96.6	99.0	0.1	0.0	1.9
Y81	100.0	100.0	100.0	5.2	0.6	9.5
A82	100.0	100.0	100.0	7.0	0.7	9.8
G83	100.0	100.0	100.0	6.9	0.7	9.3
F84	100.0	100.0	100.0	0.8	0.8	9.0
I85	52.2	5.3	11.0	8.1	2.1	6.4
L109	37.4	14.3	6.8	6.3	0.3	0.1

**Fig. 3:**
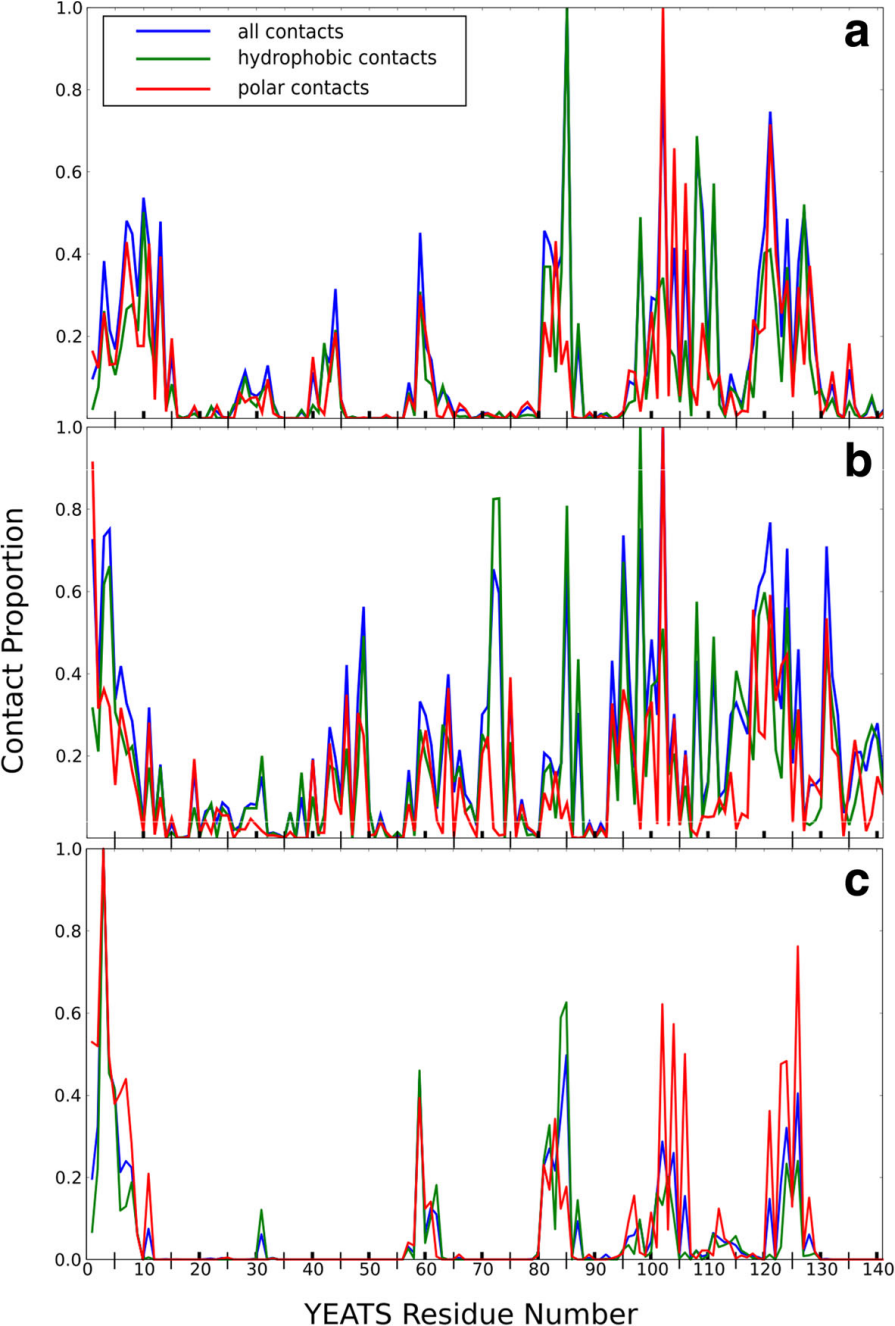
YEATS/Peptide interaction. Statistics of polar (red), hydrophobic (green) and total (cyan) YEATS residue contacts for trajectories TAMDYpt13 (**a**), TAMDYpt7 (**b**), TAMDYpt3 (**c**). Two atoms are considered in contact when their inter-atomic distance is less than 4 Å. For hydrophobic contacts, the atoms in contact have partial charges with absolute values smaller than 0.2. For polar contacts, they have partial charges with opposite signs. The partial charges come from the AMBER ff03 force field [47]. This analysis was done with a python script using the MDAnalysis module [52]

Along the re-TAMD trajectories, statistics of residue contact proportions along the YEATS domain sequence (Fig. 3) are plotted for the polar (red) and hydrophobic (blue) contacts, as well as for the total number (green) of contacts. The peptides pt13 and pt3 display contact with a subset of residues while p7 displays contact with a very wide range of residues. Therefore, the specificity of contact is greater for pt13 and pt3 than it is for pt7. Noticeably, the profiles of polar and hydrophobic contacts are mostly superimposed, except for the residue I85 for which the hydrophobic profile dominates.

**Fig. 4:**
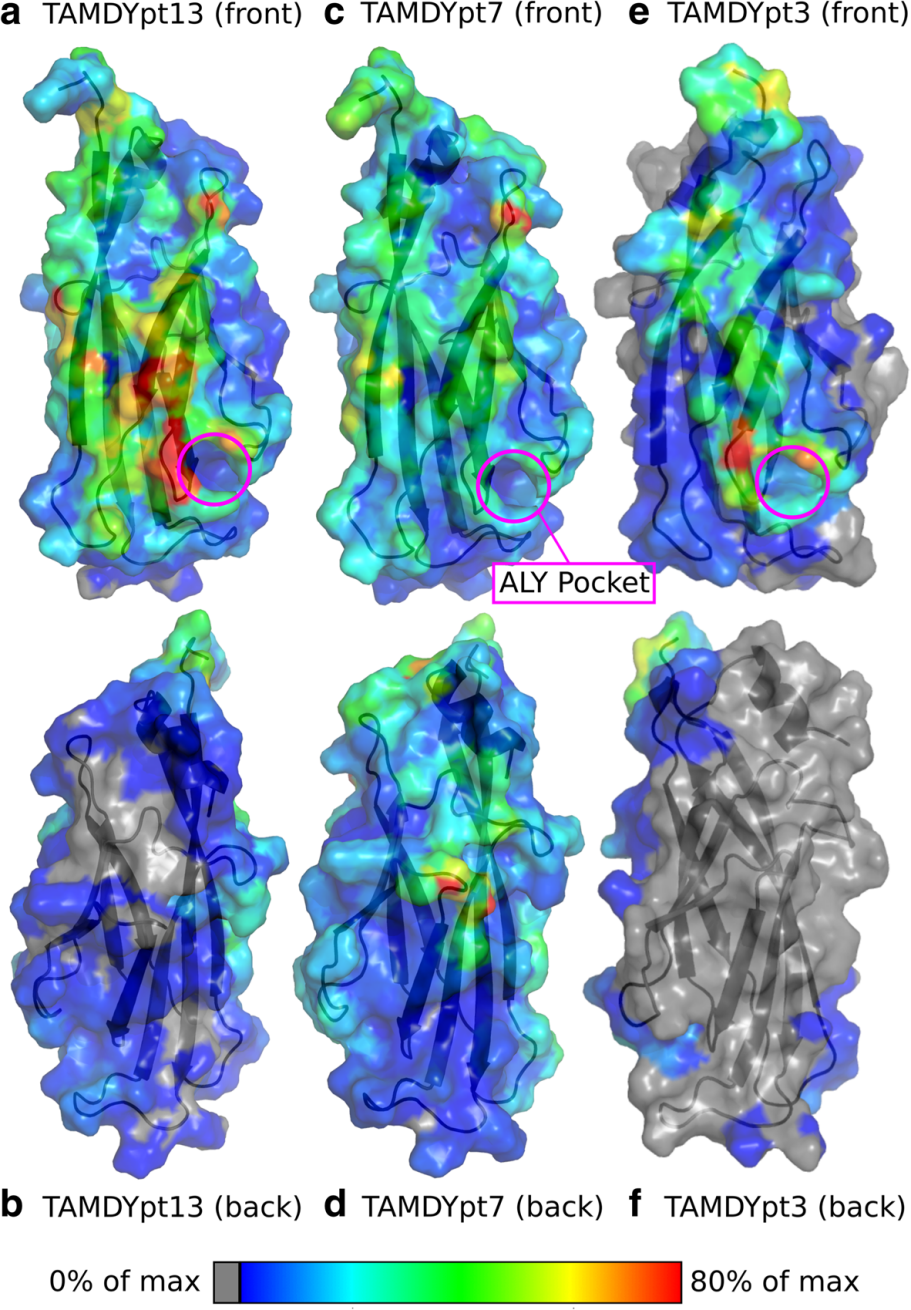
Contacts between YEATS and peptides. Surface of YEATS domain colored according to the proportion of atomic contacts with the peptide, from blue (near 0% of maximum number of contacts) to red (over 80% maximum number of contacts). The gray atoms had strictly zero contacts with the peptide throughout the trajectory. The Connolly protein surface was calculated using PyMol. The protein surface is represented with 30% transparency in order to see YEATS’ secondary structures in cartoon representation. The total number of inter-atomic contacts (between a YEATS atom and a peptide atom) for each YEATS atom was calculated along the three re-TAMD trajectories: TAMDYpt13 (**a-b**), TAMDYpt7 (**c-d**), TAMDYpt3 (**e-f**). The N terminal tails GSH bearing the Histidine tag were not drawn

**Fig. 5:**
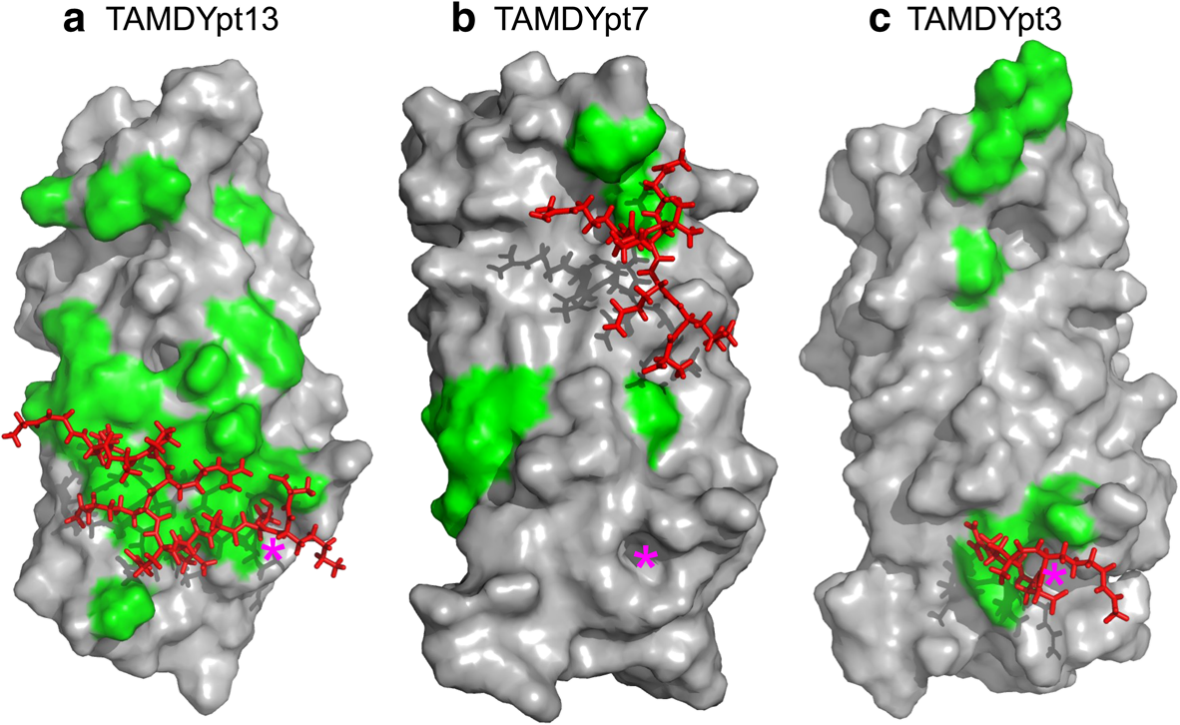
Typical peptide conformation. Typical conformations observed along TAMD trajectories TAMDYpt13 (**a**), TAMDYpt7 (**b**) and TAMDYpt3 (**c**), for the peptide and the YEATS domain. The surface of YEATS domain is shown, colored in grey, and the residues with atomic contacts larger than 50% (Table 4), are colored in green. The peptide conformation is displayed in sticks, colored in red and the position of the acK18 native binding site is marked with an asterisk

The mapping of the atomic contacts on the YEATS domain’s surface (Fig. 4) shows that the front surface, which contains the acK18 binding site, is much more sampled than the opposite surface in the case of pt13 and pt3. The peptide’s preference for the side containing the acK18 site is certainly influenced by the peptide’s initial position, but agrees with the specificity of the interaction between H3 peptide and YEATS. Also, the acK18 site is blue for all trajectories, which proves that the acK18 dissociation was complete, in agreement with Table 3, and that the peptide was mostly exploring the remaining part of the protein surface. If one compares the three different peptides, pt13 displays a more specific distribution of its atomic contact hot-spots — colored in red and corresponding to proportions greater than 80%. Indeed, most of the pt13 hot spots are located close to the acK18 binding site. Conversely, the peptide pt7 is much less specific with an almost completely blue and green surface and very few hot spots. The tripeptide pt3 displays other features as several hot spots are present, but more or less uniformly dispersed on the front surface. Also, pt3 is the peptide displaying the largest unsampled grey surface, which agrees with the observation that this peptide spends only 93% of the trajectory close to the protein surface, versus 100% and 99% for pt7 and pt13 respectively. Typical peptide conformations bound to the YEATS domain during re-TAMD trajectory are shown in Fig. 5.

**Table 4:** Protein residues displaying at least one atomic contact with the peptide in the PDB structure 2NDF or one atomic larger than 50% during the TAMD trajectories

Structure	YEATS residues
2NDF	F31 H33 H59 S61 F62 S79 G80 Y81 A82 G83 F84 I85 P87 D106 F108 L109 H110 L111
Trajectory name	YEATS residues
TAMDYpt13	S7 C8 V10 H59 A82 G83 F84 I85 P98 R102 D104 D106 F108 L109 L111 H119 L120 R121 C122
TAMDYpt7	P72 P73 P87 P98 N118 H119 L120 R121 T131 D133
TAMDYpt3	M4 A5 S7 H59 A82 G83 F84 T126

The N terminal tails GSH bearing the Histidine tag were not considered

The YEATS domain residues displaying at least one atomic contact frequency larger than 50% along re-TAMD trajectories, have been listed in Table 4. In TAMDYpt13, the number of residues with atomic contacts larger than 50% is similar to the number of contacts observed in the PDB structure 2NDF of the complex. A comparison between these two residue lists reveals that they are significantly different: the 2NDF and TAMDYpt13 lists only have about one half of their residues in common. Beside, there is a larger number of YEATS residues with atomic contacts greater than 50% for pt13 than for pt7 and pt3. The additional contacts supporting the specificity of pt13 with respect to pt7 can be clustered in three groups: (i) S7 C8 V10, located at the N terminal part on the protein side opposite to the acK18 native site, (ii) H59 A82 G83 F84 I85, located around the acK18 native site, and (iii) R102 D104 D106 F108 L109 L111, located on the surface of the *β* sheet, which may be naturally in contact with the H3 peptide when the peptide encounters the YEATS domains.

**Fig. 6:**
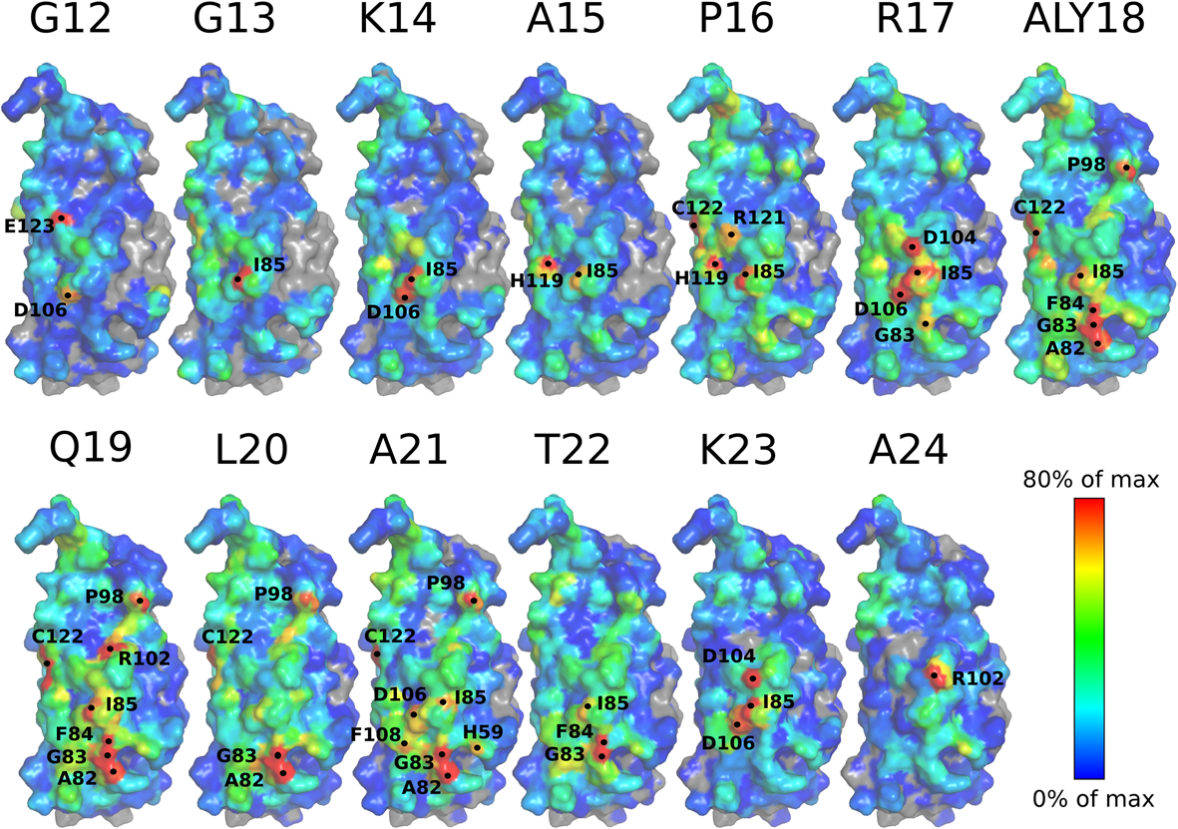
Contacts between YEATS and peptide residues. Surface of YEATS domain colored according to the proportion of atomic contacts with a given pt13 peptide residue. The total number of contacts for each YEATS atom was calculated along the TAMDYpt13 trajectory. The labels on the YEATS surface are YEATS residues displaying at least one large atomic contact with the peptide. The N terminal tails GSH bearing the Histidine tag were not drawn

In the case of pt13, the mapping of the contacts of each peptide residue to the YEATS surface (Fig. 6) reveals quite different behavior between pre-acK18 and post-acK18 residues in the H3 peptide sequence. Indeed, the post-acK18 residues Q19, L20 and A21 along with acK18 bind mostly to the YEATS residues A82, G83, F84 and I85, which are located at the entrance of the acK18 binding site. Conversely, the pre-acK18 residues G13, K14, A15, P16 and R17 bind mostly to I85, and then alternatively to D104, D106 and H119, which are more dispersed on the protein surface. Thus, the QLA motif located in the post-acK18 region seems to play an important role in the peptide sampling on the surface.

**Fig. 7:**
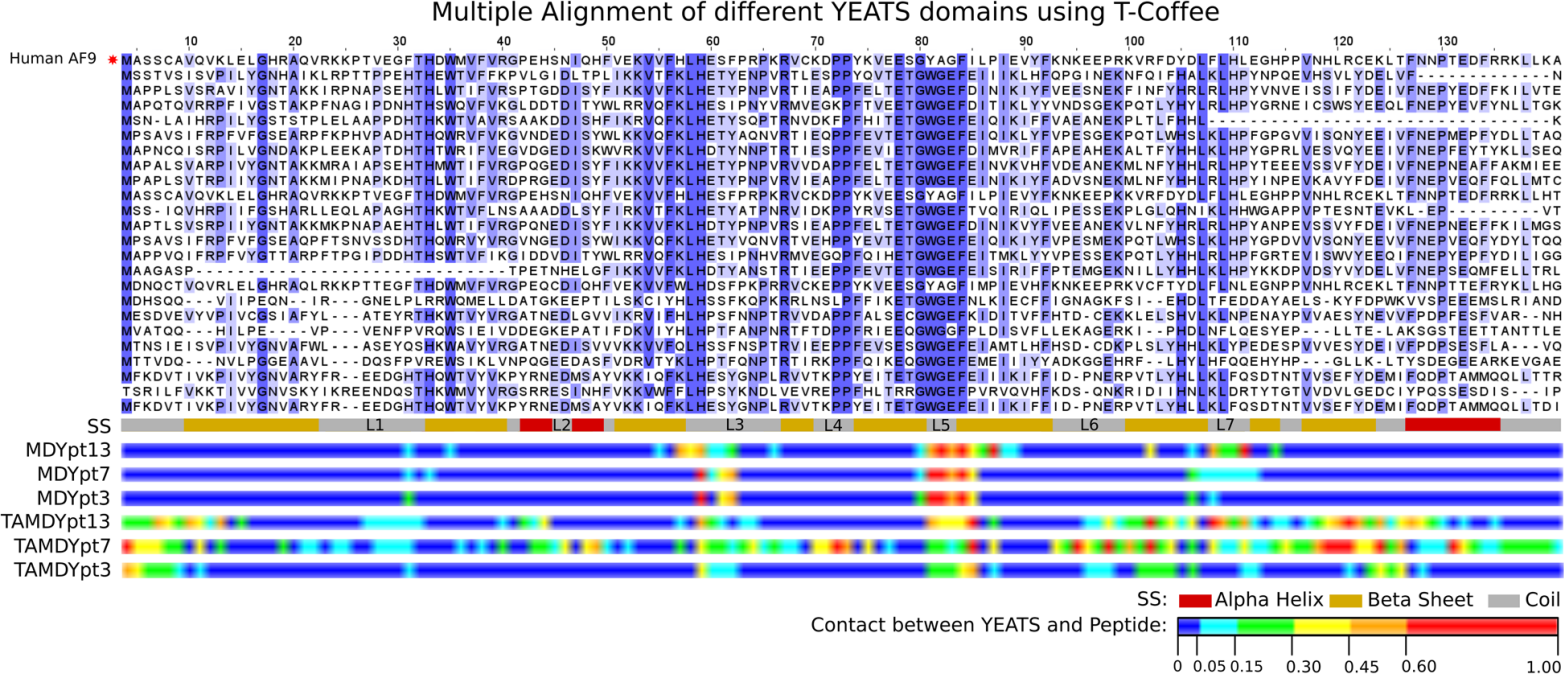
Sequence alignments. Multiple alignment between the human AF9 YEATS sequence (Uniprot ID: P42568) and other YEATS sequences (Uniprot IDs, from top to bottom: Q59LC9, Q6FXM4, Q4I7S1, Q4PFI5, Q4WPM8, Q10319, Q755P0, Q6CIV8, A2AM29, P0CM08, P53930, Q5BC71, Q7RZK7, Q6CF24, Q03111, Q99314, F4IPK2, P35189, Q9FH40, O94436, Q9CR11, Q9ULM3, O95619), colored from white to blue according to the conservation of the residue. The alignment was done using T-Coffee [54]. The columns corresponding to gaps in the human AF9 sequence were removed in order to reduce the figure width. Under the alignment, the first horizontal bar represents the secondary structure of the AF9 YEATS NMR structure (PDB 2NDF: *α* helices in red and *β* strands in orange). The following six bars represent the proportion of contact of each YEATS residue with the peptide, from blue (near 0% of maximum number of contacts) to red (over 80% maximum number of contacts). There is one bar for each trajectory: MDYpt13, MDYpt7, MDYpt3, TAMDYpt13, TAMDYpt7, TAMDYpt3. Contacts were computed with a python script using the MDAnalysis module [52]

Twenty four sequences of YEATS domains were aligned (Fig. 7) using T-Coffee [54]. The residue contact proportions are plotted below the sequence alignments. Along MD trajectories, the protein sequence 80-84, on which the acK18 binding site is centered, displays strong residue contacts. In other sequence regions, the proportion of contacts are more spread out and somehow more intense for the native peptide pt13 than for the shortened peptides pt7 and pt3. Along re-TAMD trajectories, the residue contacts are much reduced along the sequence 80-84, and spread out on other protein regions, as observed in Fig. 4 and Table 4. Among the residues displaying strong atomic contacts with pt13 (Table 4), V10, H59, P72, A82, G83, F84, P98, L109, L120, C122 are conserved in the YEATS sequence alignment (Fig. 7). The mapping obtained by re-TAMD on the AF-9 YEATS domain can thus be related to global sequence features of the YEATS family.

**Fig. 8:**
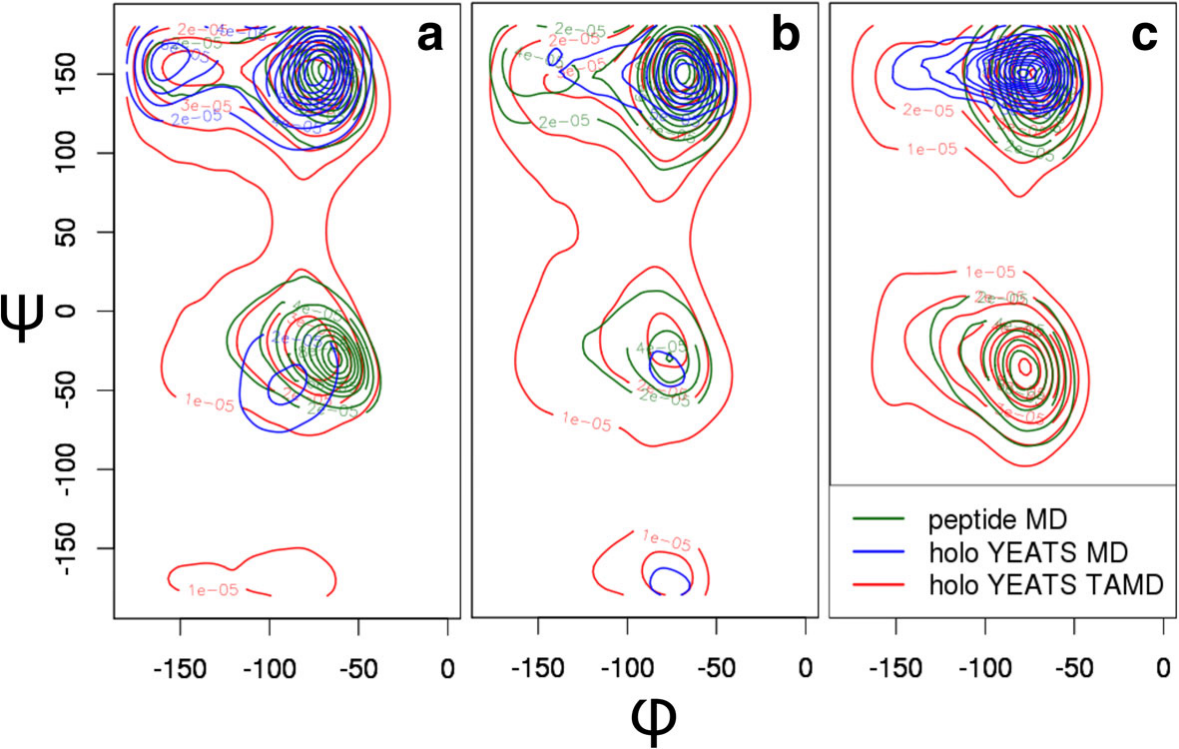
Peptide conformations. Distribution of *ϕ* and *ψ* backbone dihedral angles in the peptides pt13 (**a**) pt7 (**b**) and pt3 (**c**) along the re-TAMD trajectories [red: TAMDYpt13 (**a**), TAMDYpt7 (**b**), TAMDYpt3 (**c**)], the holo YEATS MD trajectories [blue: MDYpt13 (**a**), MDYpt7 (**b**), MDYpt3 (**c**)], and the isolated peptide MD trajectories [green: MDpt13 (**a**), MDpt7 (**b**), MDpt3 (**c**)]. The dihedral angles were computed with a python script using the MDAnalysis module [52]

The distributions of *ϕ* and *ψ* backbone angles (Fig. 8), determined on the different peptides, reveal that the sampling of the peptides in complex with the YEATS domain along re-TAMD trajectories (red) is similar to the sampling along the MD trajectories of isolated peptides (green). The re-TAMD procedure thus induces a maximal sampling of the peptide conformations, which is certainly a very positive aspect in the search of alternative binding conformations and of cryptic binding sites. Also, the large sampling observed along re-TAMD trajectories is a sign that the peptide along the surface is in a free-diffusion state along the protein surface, which is the first step of the interactions between biomolecules before the formation of the close-encounter complex [55]. In that way, the re-TAMD trajectories, which were obtained here by dissociating the peptide/protein complex, converge to the first steps of the peptide/protein association. The sampling of the peptide along the MD trajectories of the peptide/YEATS complex (blue) is more reduced than in other trajectories and this difference is most prominent in the case of MDYpt3 (Fig. 8c), in which the peptide does not sample the region of negative *Ψ* values.

## Discussion

Here, the re-TAMD approach, based on the TAMD enhanced sampling method, has been presented for performing the exploration of a receptor surface by a ligand. This approach presents several advantages. First, the energy is calculated using an all-atoms classical empirical force field, which allows a precise evaluation of inter-molecular interactions. Second, the choice of collective variables is fully open to the user, and additional collective variables could be put on the receptor, in order to study the interplay between internal dynamics and intermolecular interaction [56, 57]. In a similar way, competition of two ligands for a binding site could be studied using re-TAMD with an appropriate soft-ratcheting criterion [29]. The effects of water molecules and/or ions could also be observed. During the present work, the starting point of all re-TAMD simulations contains the peptide bound to the native site of acK18. But, starting from any point of the protein surface should be possible using a ligand pose obtained by molecular docking.

The region around the native binding site of acK18 is mainly populated for the full peptide pt13, whereas quite different surfaces are explored for shorter peptides. This agrees with the specificity of the H3 N terminal peptide, supported by the strict conservation of the primary sequence in this region. Then, according to the (*ϕ*,*ψ*) distributions, the conformational spaces sampled by the peptides along the re-TAMD trajectories are similar to the ones sampled by the isolated peptides in MD trajectories. This is in agreement with the free-diffusion step [55] for the intermolecular interactions. Furthermore, the YEATS residues displaying the most interactions with the peptide along the re-TAMD trajectories, are conserved in the sequences alignment of the YEATS domain. One should nevertheless notice that not much is experimentally known about the first steps of interactions between YEATS domain and the N terminal peptide of histone H3, so the results presented here are more evaluated to be plausible with the general knowledge on protein/peptide interaction than with a knowledge specific to YEATS/H3 interaction.

Several protein-peptide docking approaches have been developed in the literature. Several of them are based on Fast Fourier Transform (FFT) or on Normal Mode Analysis (NMA) and uses rigid docking [58]. The Normal Mode Analysis has been also used [59–62] for determining several conformations of the receptor in order to perform docking on these conformations. However, the use of FFT or of NMA does not permit to simulate the relative effect the dynamics of each interaction partner has on the other partner. By contrast, in re-TAMD simulations, the peptide as well as the protein are free to move in the force field, the peptide motions being accelerated. In that way, the peptide explores more protein surface, and one can expect that the effect of the peptide on the protein should also be enhanced.

In the present work, 100 ns of re-TAMD trajectory was recorded for each analyzed peptide. This computational load is thus smaller than that of reconnaissance or funnel metadynamics [13, 14], but much larger than that of many docking approaches [58, 63–65]. The re-TAMD approach can thus be considered to be efficient on small datasets of several dozens to a hundred ligands. This makes this method possible for virtual screening approaches focused on a ligand family. Given that for some studies, only dozens of ligands can be tested due to stringent experimental constraints, it is worth noting re-TAMD as an efficient method to map surface contacts in the context of an all-atom force field.

## Conclusions

Based on the enhanced sampling approach TAMD, the method re-TAMD has been proposed to induce a free diffusion of a peptide around a protein surface. This approach has been tested on the interaction between the N terminal peptide of the histone H3 and the reader domain YEATS. Several contact distributions are obtained on the protein surface, depending on the peptide length. Less significant contact distribution has been observed as the peptide gets shorter, putting in evidence the importance of the 13 residue peptide for the reader/histone interaction. Furthermore, the most often observed contacts involved protein residues located in conserved regions of the YEATS sequence alignment.

## Abbreviations

acK18: Acetylated lysine 18
ALY: Acetylated lysine 18 of the H3 histone
C-t: C terminal
CV: Collective variable
FFT: Fast Fourier transform
MD: Molecular dynamics
NMA: Normalmode analysis
NMR: Nuclearmagnetic resonance
N-t: N terminal
pt13: peptide with H3 histone residues 12-24
pt3: peptide with H3 histone residues 17-19
pt7: peptide with H3 histone residues 15-21
PTM: post translational modification
QM/MM: quantum mechanics/molecular dynamics
re-TAMD: Reconnaissance-TAMD
TAMD: Temperature-accelerated molecular dynamics
YEATS: Yaf9, ENL, AF9, and Sas5 family

## References

[1] Noble MEM, Endicott JA, Johnson LN (2004). Protein kinase inhibitors: insights into drug design from structure. Science.

[2] Chen Y, Scully M, Dawson G, Goodwin C, Xia M, Lu X (2013). Perturbation of the heparin/heparin-sulfate interactome of human breast cancer cells modulates pro-tumourigenic effects associated with PI3K/Akt and MAPK/ERK signalling. Thromb Haemost.

[3] Gumbart J, Roux B, Chipot C (2013). Efficient determination of protein-protein standard binding free energies from first principles. J Chem Theory Comput.

[4] Omer A, Suryanarayanan V, Selvaraj C, Singh S, Singh P (2015). Re-positioning: Predicting Novel Drug-Target Interactions of the Shelved Molecules with QM/MM Based Approaches. Adv Protein Chem Struct Biol.

[5] Cole J, Murray C, Nissink J, Taylor R, Taylor R (2005). Comparing protein-ligand docking programs is difficult. Proteins.

[6] Pagadala NS, Syed K, Tuszynski J (2017). Software for molecular docking: a review. Biophys Rev.

[7] Jaghoori MM, Bleijlevens B, Olabarriaga SD (2016). 1001 Ways to run AutoDock Vina for virtual screening. J Comput Aided Mol Des.

[8] Biesiada J, Porollo A, Velayutham P, Kouril M, Meller J (2011). Survey of public domain software for docking simulations and virtual screening. Hum Genomics.

[9] Yu W, Lakkaraju S, Raman E, MacKerell A (2014). Site-Identification by Ligand Competitive Saturation (SILCS) assisted pharmacophore modelin. J Comput Aided Mol Des.

[10] Yu W, Lakkaraju S, Raman E, MacKerell A (2015). Pharmacophore modeling using site-identification by ligand competitive saturation (SILCS) with multiple probe molecules. J Chem Inf Model.

[11] Ung P, Ghanakota P, Graham S, Lexa K, Carlson H (2016). Identifying binding hot spots on protein surfaces by mixed-solvent molecular dynamics: HIV-1 protease as a test case. Biopolymers.

[12] Ghanakota P, Carlson H (2016). Moving beyond active-site detection: MixMD applied to allosteric systems. J Phys Chem B.

[13] Limongelli V, Bonomi M, Parrinello M (2013). Moving beyond active-site detection: MixMD applied to allosteric systems. Proc Natl Acad Sci USA.

[14] Troussicot L, Guillière F, Limongelli V, Walker O, Lancelin J (2015). Funnel-metadynamics and solution NMR to estimate protein-ligand affinities. J Am Chem Soc.

[15] Söderhjelm P, Tribello G, Parrinello M (2012). Locating binding poses in protein-ligand systems using reconnaissance metadynamics. Proc Natl Acad Sci USA.

[16] Oleinikovas V, Saladino G, Cossins BP, Gervasio FL (2016). Understanding cryptic pocket formation in protein targets by enhanced sampling simulations. J Am Chem Soc.

[17] Zhu T, Cao S, Su PC, Patel R, Shah D, Chokshi HB (2013). Hit identification and optimization in virtual screening: practical recommendations based on a critical literature analysis. J Med Chem.

[18] Ferenczy GG, Keserü GM (2010). Thermodynamics guided lead discovery and optimization. Drug Discov Today.

[19] Kesarwani M, Huber E, Kincaid Z, Evelyn CR, Biesiada J, Rance M (2015). Targeting substrate-site in Jak2 kinase prevents emergence of genetic resistance. Sci Rep.

[20] Tian X, He Y, Zhou J (2015). Progress in antiandrogen design targeting hormone binding pocket to circumvent mutation based resistance. Front Pharmacol.

[21] Kozakov D, Grove LE, Hall DR, Bohnuud T, Mottarella SE, Luo L (2015). The FTMap family of web servers for determining and characterizing ligand-binding hot spots of proteins. Nat Protoc.

[22] Valsson O, Tiwary P, Parrinello M (2016). Enhancing important fluctuations: rare events and metadynamics from a conceptual viewpoint. Annu Rev Phys Chem.

[23] Maragliano L, Vanden-Eijnden E (2006). A temperature accelerated method for sampling free energy and determining reaction pathways in rare events simulations. Chem Phys Lett.

[24] Maragliano L, Fischer A, Vanden-Eijnden E, Ciccotti G (2006). String method in collective variables: Minimum free energy paths and isocommittor surfaces. J Chem Phys.

[25] Maragliano L, Cottone G, Ciccotti G, Vanden-Eijnden E (2010). Mapping the network of pathways of CO diffusion in myoglobin. J Am Chem Soc.

[26] Abrams C, Vanden-Eijnden E (2010). Large-scale conformational sampling of proteins using temperature-accelerated molecular dynamics. Proc Natl Acad Sci USA.

[27] Selwa E, Huynh T, Ciccotti G, Maragliano L, Malliavin TE (2014). Temperature-accelerated molecular dynamics gives insights into globular conformations sampled in the free state of the AC catalytic domain. Proteins Struct Funct Bioinformatics.

[28] Naveh MH, Malliavin T, Maragliano L, Cottone G, Ciccotti G (2014). Conformational changes in acetylcholine binding protein investigated by temperature accelerated molecular dynamics. PLoS ONE.

[29] Cortes-Ciriano I, Bouvier G, Nilges M, Maragliano L, Malliavin T (2015). Temperature accelerated molecular dynamics with soft-ratcheting criterion orients enhanced sampling by low-resolution information. J Chem Theory Comput.

[30] The PyMOL Molecular Graphics System. Version 1.8 Schrödinger, LLC.

[31] Peterson C, Laniel M (2004). Histones and histone modifications. Curr Biol CB.

[32] Luger K, Mader A, Robin K, Sargent D, Richmond T (1997). Crystal structure of the nucleosome core particle at 2.8 Å resolution. Nature.

[33] Dhalluin C, Carlson J, Zeng L, He C, Aggarwal A, Zhou M (1999). Structure and ligand of a histone acetyltransferase bromodomain. Nature.

[34] Tweedie-Cullen R, Reck J, Mansuy I (2009). Comprehensive mapping of post-translational modifications on synaptic, nuclear, and histone proteins in the adult mouse brain. J Proteome Res.

[35] Bannister A, Kouzarides T (2007). Regulation of chromatin by histone modifications. Cell.

[36] Kouzarides T (2007). Chromatin modifications and their function. Cell.

[37] Patel D, Wang Z (2013). Readout of epigenetic modifications. Annu Rev Biochem.

[38] Zhang Q, Zeng L, Zhao C, Ju Y, Konuma T, Zhou M (2016). Structural Insights into Histone Crotonyl-Lysine Recognition by the AF9 YEATS Domain. Structure.

[39] Zhao D, Guan H, Zhao S, Mi W, Wen H, Li Y (2016). YEATS2 is a selective histone crotonylation reader. Cell Res.

[40] Li Y, Wen H, Xi Y, Tanaka K, Wang H, Peng D (2014). AF9 YEATS domain links histone acetylation to DOT1l-mediated H3k79 methylation. Cell.

[41] Schulze J, Wang A, Kobor M (2010). Reading chromatin: insights from yeast into YEATS domain structure and function. Epigenetics.

[42] Schulze J, Wang A, Kobor M (2009). YEATS domain proteins: a diverse family with many links to chromatin modification and transcription. Biochem Cell Biol.

[43] Audia J, Campbell R (2016). Histone modifications and cancer. Cold Spring Harbor Perspect Biol.

[44] Erb M, Scott T, Li B, Xie H, Paulk J, Seo H (2017). Transcription control by the ENL YEATS domain in acute leukaemia. Nature.

[45] Wan L, Wong H, Li Y, Lyu J, Xi Y, Hoshii T (2017). ENL links histone acetylation to oncogenic gene expression in acute myeloid leukaemia. Nature.

[46] Salomon-Ferrer R, Case DA, Walker RC (2013). An overview of the Amber biomolecular simulation package. WIREs Comput Mol Sci.

[47] Duan Y, Wu C, Chowdhury S, Lee M, Xiong G, Zhang W (2003). A point-charge force field for molecular mechanics simulations of proteins based on condensed-phase quantum mechanical calculations. J Comput Chem.

[48] Khoury G, Thompson J, Smadbeck J, Kieslich C, Floudas C (2013). Forcefield ptm: Ab initio charge and AMBER forcefield parameters for frequently Oc- curring post-translational modifications. J Chem Theory Comput.

[49] Phillips J, Braun R, Wang W, Gumbart J, Tajkhorshid E, Villa E (2005). Scalable molecular dynamics with NAMD. J Comput Chem.

[50] Perilla J, Beckstein O, Denning E, Woolf T (2011). Computing ensembles of transitions from stable states: Dynamic importance sampling. J Comput Chem.

[51] Perilla J (2015). Computing ensembles of transitions with molecular dynamics simulations. Methods Mol Biol.

[52] Michaud-Agrawal N, Denning E, Woolf T, Beckstein O (2011). MDAnalysis: a toolkit for the analysis of molecular dynamics simulations. J Comput Chem.

[53] Schrödinger LLC. The PyMOL Molecular Graphics System, Version 1.8. 2015.

[54] Notredame C, Holm L, Higgins DG (1998). COFFEE: an objective function for multiple sequence alignments. Bioinformatics.

[55] Spaar A, Dammer C, Gabdoulline R, Wade R, Helms V (2006). Diffusional encounter of barnase and barstar. Biophys J.

[56] Ma B, Nussinov R (2004). Release factors eRF1 and RF2: a universal mechanism controls the large conformational changes. J Biol Chem.

[57] Bakan A, Bahar I (2009). The intrinsic dynamics of enzymes plays a dominant role in determining the structural changes induced upon inhibitor binding. Proc Natl Acad Sci U S A.

[58] Porter KA, Xia B, Beglov D, Bohnuud T, Alam N, Schueler-Furman O (2017). ClusPro PeptiDock: efficient global docking of peptide recognition motifs using FFT. Bioinformatics.

[59] Moroy G, Sperandio O, Rielland S, Khemka S, Druart K, Goyal D (2015). Sampling of conformational ensemble for virtual screening using molecular dynamics simulations and normal mode analysis. Future Med Chem.

[60] Bakan A, Bahar I. Computational generation inhibitor-bound conformers of p38 MAP kinase and comparison with experiments. Pac Symp Biocomput. 2011:181–92. https://www.ncbi.nlm.nih.gov/pubmed/21121046. 10.1142/9789814335058_0020 PMC4782186 21121046

[61] Leis S, Zacharias M (2011). Efficient inclusion of receptor flexibility in grid-based protein-ligand docking. J Comput Chem.

[62] Sperandio O, Mouawad L, Pinto E, Villoutreix BO, Perahia D, Miteva MA (2010). How to choose relevant multiple receptor conformations for virtual screening: a test case of Cdk2 and normal mode analysis. Eur Biophys J.

[63] Marcu O, Dodson EJ, Alam N, Sperber M, Kozakov D, Lensink MF (2017). FlexPepDock lessons from CAPRI peptide-protein rounds and suggested new criteria for assessment of model quality and utility. Proteins.

[64] Yu J, Andreani J, Ochsenbein F, Guerois R (2017). Lessons from (co-)evolution in the docking of proteins and peptides for CAPRI Rounds 28-35. Proteins.

[65] van Zundert GCP, Rodrigues JPGLM, Trellet M, Schmitz C, Kastritis PL, Karaca E (2016). The HADDOCK2.2 webserver: User-friendly integrative modeling of biomolecular complexes. J Mol Biol.

